# Nuclear envelope-distributed *CD147* interacts with and inhibits the transcriptional function of *RING1* and promotes melanoma cell motility

**DOI:** 10.1371/journal.pone.0183689

**Published:** 2017-08-23

**Authors:** Junchen Chen, Cong Peng, Li Lei, Jianglin Zhang, Weiqi Zeng, Xiang Chen

**Affiliations:** 1 Department of Dermatology, Xiangya Hospital, Central South University. Changsha, Hunan, P.R. China; 2 Hunan Key Laboratory of Skin Cancer and psoriasis, Central South University. Changsha, Hunan, P.R. China; Duke University School of Medicine, UNITED STATES

## Abstract

Melanoma accounts for nearly 80% of all deaths associated with skin cancer.*CD147* plays a very important role in melanoma progression and the expression level may correlate with tumor malignancy. *RING1* can bind DNA and act as a transcriptional repressor, play an important role in the aggressive phenotype in melanoma. The interactions between *CD147* and *RING1* were identified with a yeast two-hybrid and *RING1* interacted with *CD147* through the transmembrane domain. *RING1* inhibits *CD147*’s capability promoting melanoma cell migration. In conclusion, the study identified novel interactions between *CD147* and *RING1*, recovered *CD147* nuclear envelope distribution in melanoma cells, and suggested a new mechanism underlying how cytoplasmic *CD147* promotes melanoma development.

## Introduction

Although melanoma represents only 4% of all skin cancers, it accounts for nearly 80% of all deaths associated with skin cancer [1–3]. Accumulating data show that melanoma progression involves a series of genetic and environmental changes, including enhanced anaerobic glycolysis, microenvironment remodeling caused by matrix metalloproteinase (MMPs), genome epigenetic modification changes and angiogenesis.

*CD147*,a type I integral membrane receptor protein that belongs to the immunoglobulin superfamily[4], is a widely expressed integral plasma membrane glycoprotein that stimulates the secretion of extracellular MMPs, also known as a membrane extracellular matrix metalloproteinase inducer (*EMMPRIN/Basigin*) [5–7]. It consists of two Ig-like extracellular domains, a transmembrane domain and a cytoplasmic domain. Its transmembrane domain contains highly conserved glutamic acid residues and leucine zipper-like sequences, allowing it to interact with multiple signaling proteins of other membrane proteins[8]. The expression level of *CD147* in many tumor cells may correlate with tumor malignancy[9–11].

*CD147* is primarily distributed on the cellular membrane in multiple types of human tissues, and it has been shown to regulate the function of many membrane proteins, such as *MCT*s[12, 13], *GLUTs* [14], *ABCGs*[15, 16] and *Integrins* [17]. To date, most investigations on *CD147* have focused on its cellular membrane functions[18, 19]. Interestingly, we have found that in metastatic malignant melanomas, *CD147* is largely redistributed in cellular inner membrane systems [20], such as the mitochondrial membrane[21], ER[22] and nuclear envelope. *CD147* interacts with Ubiquinone Oxidoreductase Subunit S6 (*NDUFS6*) on the mitochondrial membrane and regulates the oxidative phosphorylation process and is required for melanoma cell glucose metabolism. It also interacts with Calcium Modulating Ligand (*CAMLG*) in the endoplasmic reticulum (ER) and regulates calcium homeostasis in melanoma cells.

PcG proteins also play an important role in the aggressive phenotype in melanoma[23]. The PcG proteins form two distinct complexes: EED-EZH2 and the PRC complex[24]. *RING1* is part of the *PRC1* complex and contributes to PcG protein function. *RING1* proteins belong to the *RING* finger family of proteins characterized by a *RING* domain, which is a zinc-binding motif related to the zinc finger domain. *RING1* can bind DNA and act as a transcriptional repressor[25].

The matrix Gla protein (MGP) is initially isolated from the bone tissue and is also expressed in kidney, lung, heart, cartilage and vascular smooth muscle cells. [26]. It is up-regulated in a variety of tumors, including ovarian, breast, urogenital and skin tumors[27–31].

Our preliminary data from a yeast two-hybrid system have shown that several nuclear proteins interact with *CD147* in vitro, including *RING1*. In this study, we investigated the details of the interactions and functions of nuclear protein *RING1* in the regulation of downstream gene transcription.

## Materials and methods

### Cell culture

The human malignant melanoma cell line A375 was obtained from the American Type Culture Collection. The A375 cells expressing recombinant plasmid SUPER/CD147 short hairpin RNA (shRNA) (A375 ShCD147) or recombinant plasmid SUPER/RING1 short hairpin RNA (shRNA) (A375 ShRING1) or empty vector (A375 EV). These cells were maintained in RPMI 1640 medium (Thermo Scientific, MA, USA) supplemented with 10% FBS (Thermo Scientific, MA, USA).The 293T cell line was purchased from Clontech (Clontech, CA, USA) and grown in Dulbecco’s modified Eagle’s medium(DMEM) supplemented with 10% FBS. All cell lines were maintained in a humidified 5% CO2 atmosphere at 37°C.

### PCR and cloning

Wild type *CD147* and deletion mutants were fused to a transcript encoding Myc, and wild type RING1 was fused to a transcript encoding Flag. Deletion mutants of *CD147* and the full length (FL) RING1 promoter were amplified by PCR.The pcDNA4ToA and pcDNA3ToA plasmids (Promega, WI, USA) were double digested with the same restriction enzymes. Both PCR products and plasmids were recovered from 1.5% agarose gels and ligated using T4 ligase (Takara Bio, Otsu, Japan) to yield pcDNA4ToA- CD147 and pcDNA3ToA- RING1. After a 1 h of incubation at room temperature, plasmids of colonies grown on ampicillin-containing LB medium. Positive colonies were identified by PCR and direct sequencing. PCR was performed using the primers listed in S1 Table.

### RNA analysis

Total RNA was isolated from cells with TRIzol (Invitrogen, CA, USA) according to the manufacturer’s instructions. RNA was dissolved in 60 μl of DEPC-treated water. Reverse transcription and real-time PCR (RT-PCR) were performed with 2 μg of total RNA using Reverse Transcriptase AMV (Takara Bio, Otsu, Japan) and Premix Ex TaqTM II kit (Takara Bio, Otsu, Japan) according to the manufacturer’s instructions. The primers are listed in S1 Table.

### The yeast two-hybrid screen

A human fetal cDNA library and a yeast two-hybrid(Y2H) system were purchased from Clontech (Cat. 637242). Wild type *CD147* was reverse transcribed and the cDNA amplified and inserted into pGBKT7 to yield pGBKT7-*CD147*. This recombinant plasmid and the library plasmids containing the human fetal brain cDNA library were co-transformed into the AH109 yeast strain. After screening according to the manufacturer’s instructions, the prey plasmids were purified from positive clones and yeast co-transformed with pGBKT7-*CD147* and prey plasmid to verify the interaction. These transformants with both bait and prey plasmids were plated on synthetic defined dropout medium.Transformants with non-interacting protein pairs were able to grow on media lacking leucine and tryptophan (SD/-Leu/-Trp or -2 SD medium), while only positive clones containing interacting prey and bait proteins were able to grow on dropout medium lacking tryptophan, leucine, histidine, and adenine (SD/-Leu/-Trp/-His/-Ade or -4 SD medium). The positive colonies were lysed and subjected to the ortho-nitrophenyl-b-D-galactopyranoside (ONPG) assay to verify the interaction. Finally, confirmed positive colonies were picked and individual plasmids amplified, purified, and analyzed by sequencing.

### Immunoprecipitation and Western blotting

Briefly, according to the TurboFect Transfection manual (Thermo Scientific). When 293T cells grew to 70–80% were co-transfected with plasmids for 36 h. After the cells were lysed and were centrifuged at 12,000 rpm for 15 min and the supernatant protein concentration was determined by a BCA protein assay (Santa Cruz, CA, USA) for co-immunoprecipitation (CO-IP). Proteins were incubated with magnetic beads linked to anti-Flag (Sigma), anti-Myc (Santa Cruz), anti-CD147 (Abcam, Cambridge, UK), or control IgG and then with protein A/G beads (Sigma)for overnight 4°C. All of the beads beads were washed with phosphate-buffered saline (PBS), added to 40 μl lysis buffer plus 10 μl of loading buffer and incubated at 95°C for 5 min. The proteins were separated by SDS–PAGE and electroblotted onto to polyvinylidene difluoride (PVDF) membranes (Millipore, MA, USA). The immunoblots were probed with anti-Myc (Santa Cruz, 1:1000), anti-Flag (Sigma, 1:10,000), anti-CD147 (Abcam, 1:1000), anti-RING1 (Abcam, 1:100), or anti-β-actin (Sigma, 1:10,000) overnight at 4°C being blocked in medium containing 5% non-fat milk,TBS (25 mM Tris balanced to pH 8.0, 150 mM NaCl) and 0.1% Tween 20. Immunolabeled proteins were then treated with goat anti-mouse antibody (Sigma, 1:10,000) for 1 hour at room temperature and subsequently treated with goat anti-mouse IgG-HRP antibody or a goat anti-rabbit antibody (Sigma, 1:10,000). The immunoblots were developed with an enhanced chemiluminescence detection system (Bio-Rad, CA, USA).

### Immunofluorescence staining

The transfected A375 cells or the A375 cells without transfection were grown on glass coverslips. After washing with PBS, both the transfected A375 cells or the A375 cells without transfection were fixed, permeabilized and were incubated with 1% BSA in PBS (adjusted pH value to 7.2–7.4) for 1 h at room temperature. The cells were then incubated with with mouse anti-Myc (Santa Cruz, 1:100) and rabbit anti-Flag (Sigma,1:100) or mouse anti-CD147 (Abcam, 1:100) and rabbit anti-RING1 (Santa Cruz,1:50) in a humidified box for 4°C overnight. Immunolabeling was visualized by incubation in Alexa Fluor-conjugated secondary antibodies (Invitrogen, 1:200) for 1 h at room temperature. The slides were covered with Vectashieldr Mounting medium containing DAPI (Vector Laboratories, CA, USA) and viewed under an inverted fluorescence microscope (Leica, Solms, Germany).

### Design and transfection of siRNA targeting *CD147* and *RING1*

The small interfering RNAs (siRNAs) were designed using siDirect2.0 software (http://sidirect2.rnai.jp/) and synthesized by GenePharma Company (Shanghai, China). Two siRNAs targeting *CD147* mRNA, si-1039 and si-1207, and one siRNA targeting *RING1* mRNA (si-1039 sense 5’-ACAUAAAAGCACAAAAAUGGC-3’ and anti-sense 5’-CAUUUUUGUGCUUUUAUGUUU-3’, si-1207 sense 5’-CAUACACUUCCUUCUUUUUUA-3’ and anti-sense 5’-AAAAAGAAGGAAGUGUAUGAU-3’). A scrambled siRNA (GenePharma) that does not target any gene was used as a negative control siRNA. A375 cells were transfected with siRNA and transfection reagent according to the manufacturer’s instructions. Briefly, A375 cells were seeded in 35mm dishes at a density of 2 × 105 cells/well with 2 ml of RPMI 1640 and transfected with 75 pmol si-1039 or si-1207 using 7.5 μl/dish of Lipofectamine RNAiMAX (Invitrogen)according to the manufacturer’s instructions. After 72 h, cells were collected for RNA and protein to determine the efficiency of *CD147* knockdown by western blotting or RNA Analysis.

## Results

### Identification of *CD147* that interacts with *RING1*

**Fig 1:**
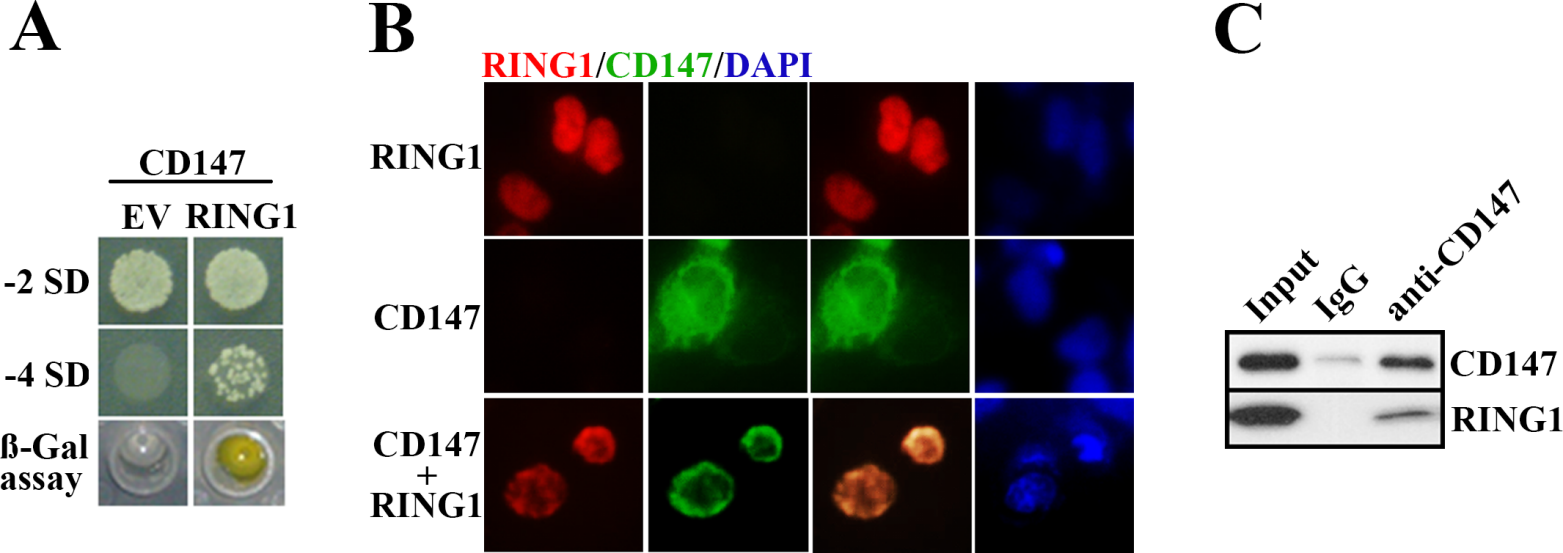
The interaction between *RING1* and *CD147* in vitro. *RING1* colocalizes with *CD147* proteins on the nuclear envelope of melanoma cells. (A) Clones were abundant in both -2 SD and -4 SD culture media when the yeast cells were transfected with *RING1* and *CD147* compared with yeast cells transfected with *CD147* and EV. In these colonies, the ONPG-containing medium became a yellow color, thus confirming the interaction between *RING1* and *CD147*. By comparison, the yeast co-transfected with *CD147* and EV produced no viable colonies, and the OPNG assay was negative. (B) Exogenous *CD147* and *RING1* were co-localized in regions surrounding the nuclear envelope in 293T cells, as identified by immunofluorescence staining.(C) 293T cells were harvested and subjected to immunoprecipitation (IP) with mouse normal IgG and anti-CD147 Ab. The membranes were further probed with the indicated antibodies.

To identify the cellular targets or possible interactive molecules with *CD147*,we used Y2H screening. The yeast strain AH109 was transformed with two proteins (namely bait and prey) are fused to the activation domain (AD) or the DNA-binding domain (BD) of the Gal 4 transcription factor and, if interaction occurs, Gal4 activates a set of reporter genes. The open reading frame (ORF) of *CD147* was cloned in frame with the GAL4 DNA-BD of pGBKT7. If the *CD147* could interact with some proteinsit allows yeast to grow in dropout media. Among the positive clones, we focused on *RING1* as a possible binding partner with *CD147*. To identify the result, the yeast strain AH109 was transformed with *CD147* and either EV or *RING1* and plated on dropout medium. Fig 1A shows that only the presence of *CD147* and *RING1* allowed for growth of the transformants on -4SD medium. To confirm the interaction, the positive colonies were subjected to an ONPG assay (Fig 1A). To verify the results of the Y2H screen, we performed immunofluorescence staining and CO-IP. 293T cells expressing exogenous *CD147* and *RING1* stained with PE-conjugated anti-RING1 (red) and FITC-conjugated anti-*CD147* (green) display partial co-localization in the emphasized regions surrounding the nuclear envelope (Fig 1B). After the 293 T cells were transfected with CD147-MYC and Flag-RING1 plsamids, the cell lysates were resolved by 10% SDS-PAGE and probed for the presence of *CD147*or *RING1*using anti-CD147 protein mAb, as evidenced by CO-IP, thereby indicacing a *CD147*-*RING1* interaction(Fig 1C). These results confirmed that *CD147* was able to interact with *RING1*.

### *CD147* colocalizes with *RING1* dependent on transmembrane domain

**Fig 2:**
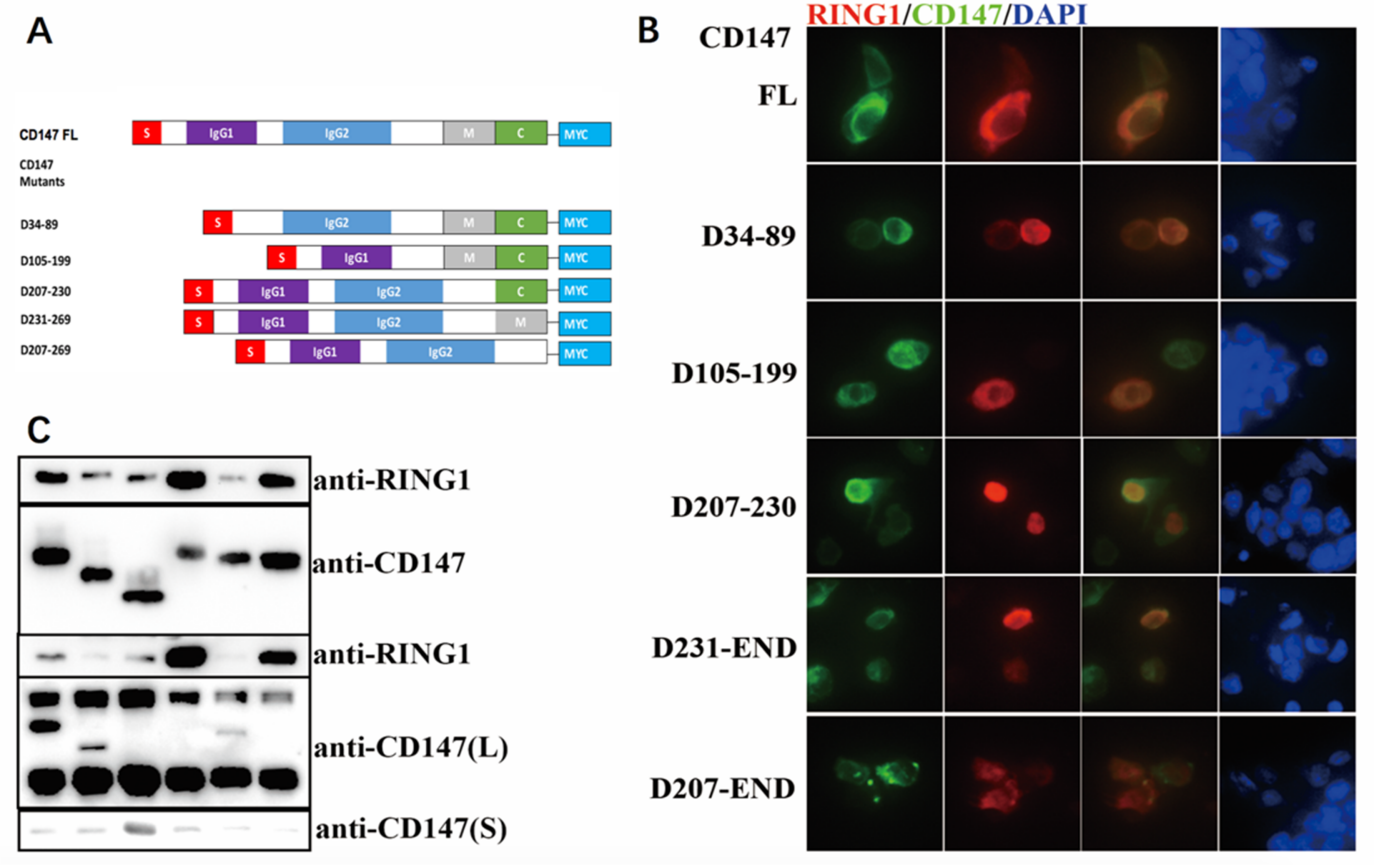
*CD147* colocalizes with *RING1* dependent on transmembrane domain. (A) An illustration of the *CD147* mutants (left) used in CO-IP and immunofluorescence experiments;34–87: 1st IgG (IgG1); 105–199: 2nd IgG (IgG2); 207–230: transmembrane (M); 231–269: cytoplasmic domain(C). (B) The A375 cells co-transfected with Flag-*RING1*and different *CD147*-MYC deletion mutants were stained with fluorophore-conjugated anti-Flag (red) and anti-Myc (green). The *CD147* mutants without the transmembrane domain (D207-230 and D207-269) showed no co-localization with *RING1*. (C) Cells (293T) were co-transfected with plasmids encoding Flag-RING1 and different CD147-MYC deletion mutants. Transfected 293T cells were subjected to IP with anti-Flag Ab. The immunoprecipitates and the whole cell lysates were further analyzed by IB with anti-Flag Ab and anti-Myc Ab.

We previously indicated that *CD147* was able to interact with *RING1*. To further investigate the influence factors of the colocalization, we constructed the mutants of *CD147*. The full-length protein is composed of two extracellular immunoglobulin (Ig) domains with a total of 185 amino acids (aa), 39 aa cytoplasmic domains and 24 aa transmembrane domains (Fig 2A). Each domain can interact with different proteins. We synthesized five different *CD147* deletion mutants and then the plasmids as the indicated images shown were co-transfected into the eukaryotic expression plasmid pcDNA4ToA, which adds the Myc epitope to the C-terminus of the inserted gene. The expression was detected by western blotting (Fig 2C). By Immunofluorescence staining observations it was found that the co-localization of the proteins is evident in all cells, as shown by the yellow region on the combined image, except in those expressing the deletion mutant D207-269 (lacking transmembrane and intracellular domains), so as to devastate that the transmembrane region is essential for *RING1* and *CD147* interaction (Fig 2B).

### *CD147* gene knockdown in A375 cells affected downstream gene expressions

**Fig 3:**
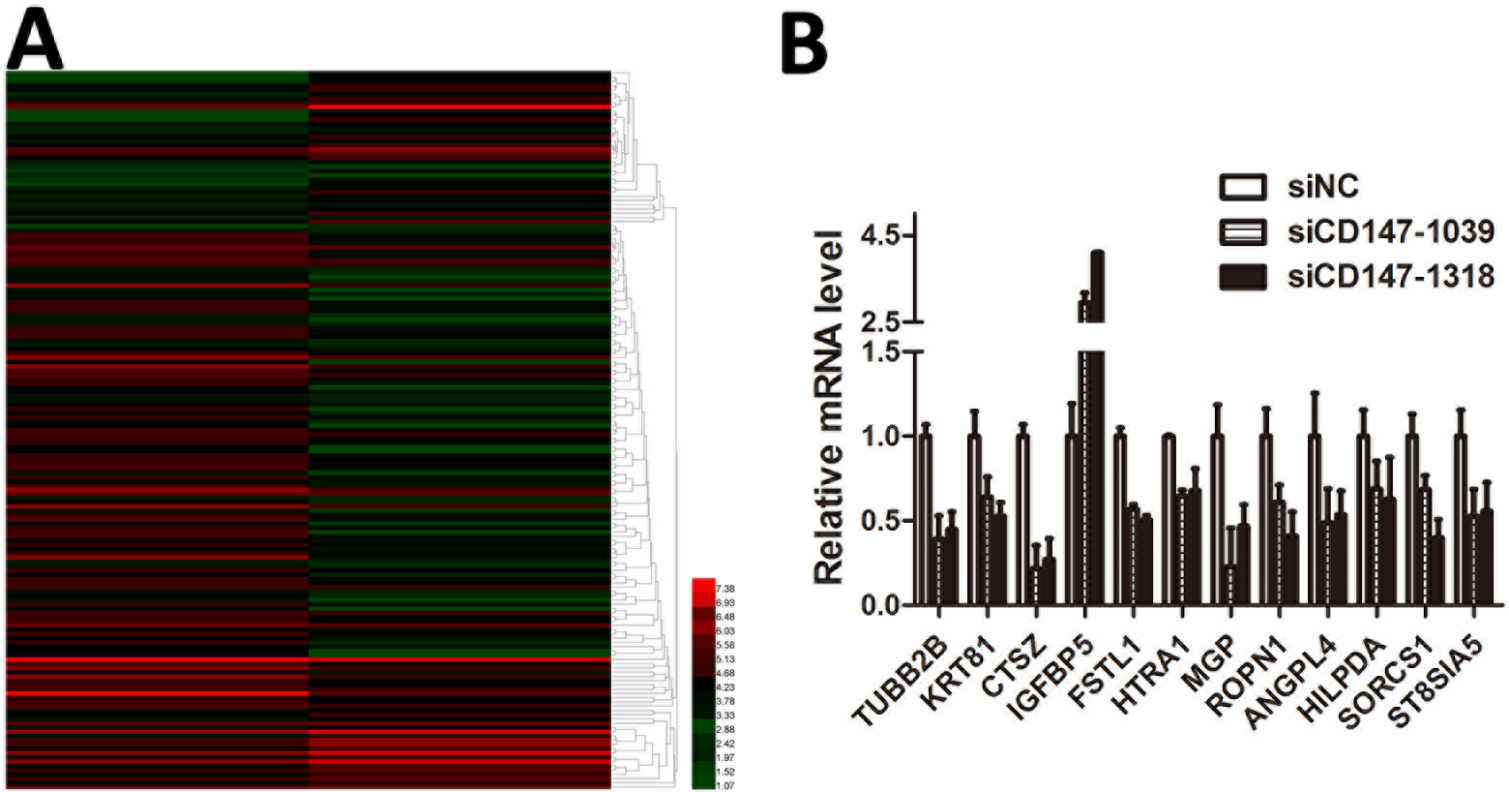
*CD147* gene knockdown in A375 cells affected many gene translation pathways, thus leading to the differential expression of hundreds of genes. (A) Heatmap representing the expression levels of genes; red (normal) and green (siCD147) colors indicate the up- and down-regulated genes. Transcriptome analysis demonstrated 67 up-regulated and 115 down-regulated genes in A375 cells transfected with siCD147. (B) Several genes referred to in A were shown to be up- or down-regulated in A375 cells by RT-PCR The result was consistent with the transcriptome analysis.

Through siRNA interference technology, we knocked down the *CD147* gene in A375 cells, and used gene chip technology to investigate the effect of *CD147* on downstream gene transcriptome. By examining the heatmap (Fig 3A), we found that after knockdown of *CD147*, transcriptome analysis showed at least 67 up-regulated genes and 115 down-regulated genes(S2 Table). To further validate the results, RT-PCR was performed, and the results were consistent with the transcription group sequencing (Fig 3B).

### Reduced *RING1* expression reverses the decrease in *MGP* expression caused by *CD147* depletion

**Fig 4:**
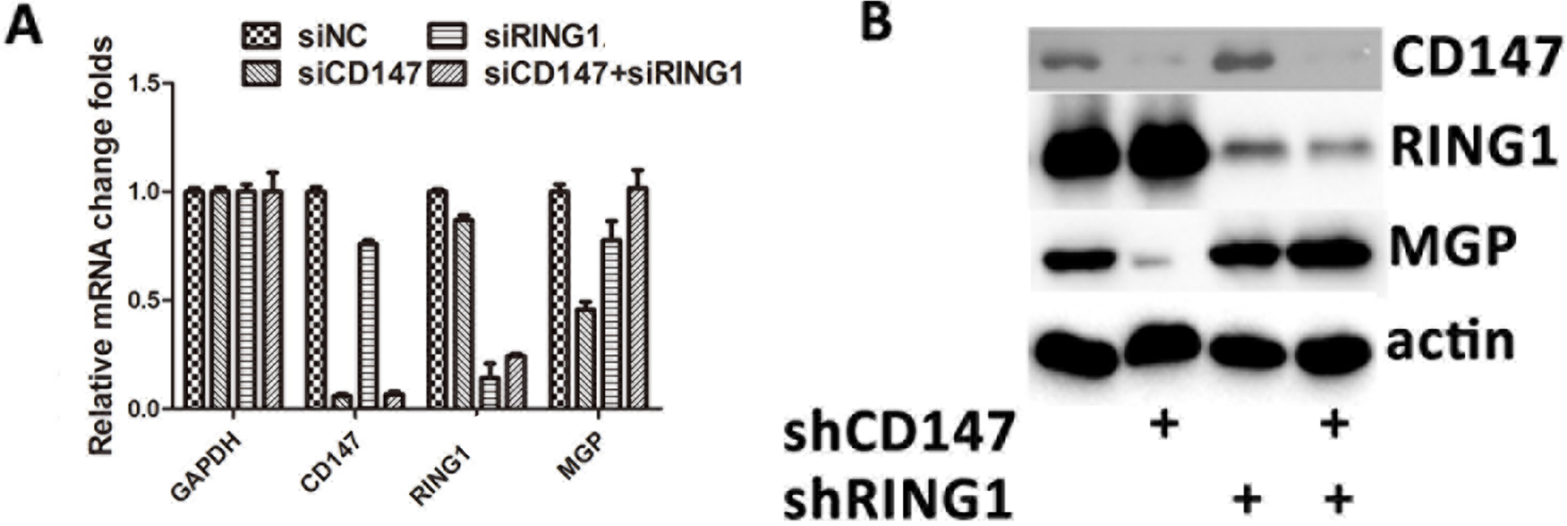
Reduction of *MGP* caused by *CD147* depletion was rescued by *RING1* depletion. (A) *CD147* knockdown in A375 cells (SiCD147-A375) led to the decreased expression of MGP mRNA. Knockdown of *RING1* in siCD147-A375 cells rescued the expression of MGP mRNA. (B) *RING1* knockdown in siCD147-A375 cells rescued the expression of MGP, as shown by western blotting analysis.

In A375 cells (SiCD147-A375), *CD147* knockdown resulted in a decrease in MGP mRNA expression (Fig 4A), whereas knockdown of *RING1* in SiCD147-A375 cells rescued the expression of *MGP* mRNA. Furthermore, to verify the change in mRNA, *RING1* knockdown in siCD147-A375 was confirmed by western blot analysis to restore the expression of *MGP* (Fig 4B).

### *RING1* inhibits CD147’s capability promoting melanoma cell migration

**Fig 5:**
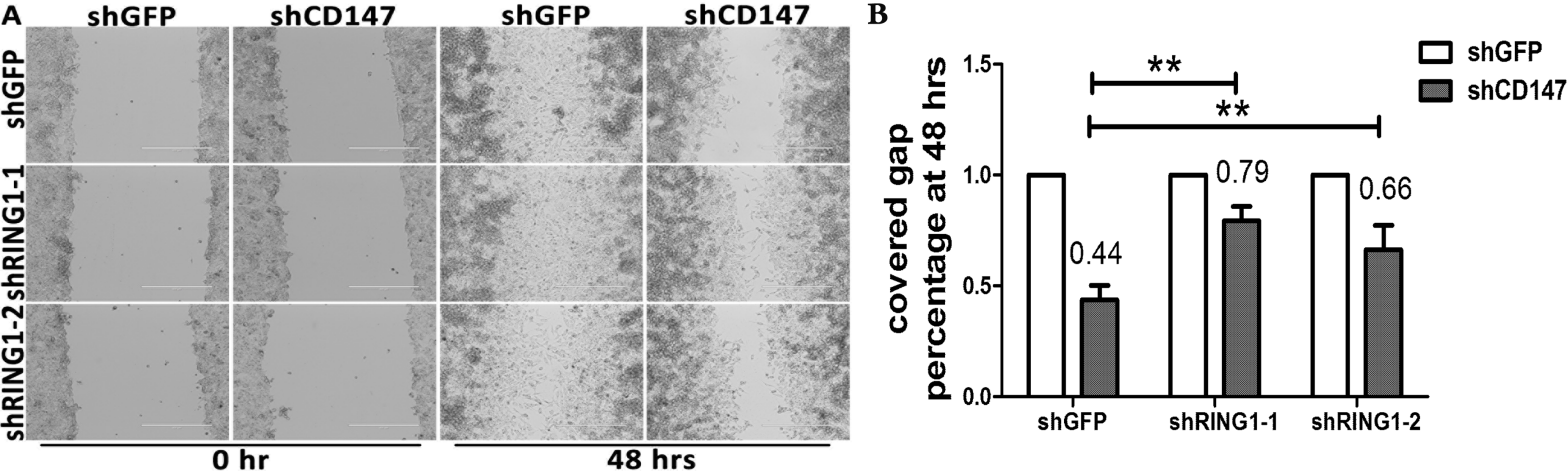
*RING1* depletion led to impaired migration ability in A375 cells transfected with shCD147. (A) Cell migration was assessed with a wound healing assay by measuring the distance between the wound edges at the indicated time points in both A375 cells transfected with shCD147 and A375 cells transfected with shCD147 and shRING1. (B) Five random experiments were performed. The gap was measured by Image plus 6.0 software. The ratio of the gap at 48 h to 0 h was analyzed using GraphPad software; t-tests were performed, and the p values are represented by a double asterisk (**, p<0.01).

Cell migration was assessed with a wound healing assay, and in the A375 cells transfected with shCD147 and A375 cells transfected with shCD147 and shRING1.The effect of depletion of *CD147* is significant in invasive capacities of both of the co-transduced tumors cell by comparing shGFP-shGFP (upper lane, third picture) and shGFP-shCD147 (upper lane, fourth picture)(Fig 5A). With CD147, cells completely rescued the gap at 48 hrs, while only 44% gap can be rescued under *CD147* depletion condition. Comparing to shGFP-shCD147, shRING1-shCD147 show 79% and 66% rescued gaps at two different shRNA targets(Fig 5B). That suggests depletion *RING1* under *CD147* knockdown background can rescue the cell motility.

## Discussion

*CD147* has been found to be related to cancer development because of its ability to regulate multiple different pathways, including up-regulating *MMPs* levels, recruiting *MCT*s to membrane localizations, and enhancing glycolysis by promoting expression of *GLUTs* [32–34]. Almost all of the functions of *CD147* that have been investigated to date have been associated with the membrane portion of *CD147*[35]. We have previously reported that *CD147* distributes to the cytoplasm in certain metastatic melanomas and have identified novel functions of *CD147* in the endoplasmic reticulum (ER), including maintaining both calcium homeostasis and mitochondrial function [21, 36]. This study identified the nuclear envelope distribution of *CD147* in melanoma and highlights a novel mechanism of *CD14*7 in controlling melanoma metastasis.

A nuclear envelope protein that regulates transcription has been identified. Lamin is a typical nuclear protein with a role in maintaining nuclear structure. A recent investigation has shown that lamin on the nuclear envelope recruits certain transcriptional factors and inhibits their transcriptional activities by sequestering them at the nuclear envelope and may preventing them from interacting with DNA[37]. We found that *CD147* plays a similar role on the nuclear envelope. *RING1* expression in cells with *CD147* knockdown showed a clear distribution throughout the nucleus but *RING1* recruitment was observed after co-expression with *CD147*. *CD147* is a ´binding protein´ that regulates many membrane functional proteins by recruiting them to the membrane. On the basis of our previous results, *CD147* binds many partners through its transmembrane domain[19]. This finding suggests that the binding may be a lyophobic association, which is less specific than a protein-protein interaction based on domain structure. Thus, *RING1* may not be the only target of *CD147* on the nuclear envelope. In future work, we are planning to use a biotin-based method to identify the potential targets.

Epigenetic modification changes have been connected with cancer development [38]. *RING1* can modify histone ubiquitination in the promoter regions of certain downstream target genes [39]. In our RNA-seq data, the numbers of genes expressed changed when *CD147* was depleted by siRNA, but the expression of only *MGP* and *ANGPTL4* (ANGPTL4 data not shown) genes was rescued when *RING1* was co-depleted. We presume that this difference might have been caused by insufficient sensitivity of the RNA-seq. Many downstream target genes have been identified and will be verified individually through real-time PCR in our future studies.

## Conclusions

In conclusion, our study identified novel interactions between *CD147* and *RING1*, recovered *CD147* nuclear envelope distribution in melanoma cells, and suggested a new mechanism underlying how cytoplasmic *CD147* promotes melanoma development.

## Supporting information

S1 Table The primers of RNA analysis. (DOCX)

S2 Table The mRNAs of *CD147* gene knockdown or not in A375 cells by microarray meta-analysis. (XLSX)
