# Supplementary material for: Nuclear envelope-distributed CD147 interacts with and inhibits the transcriptional function of RING1 and promotes melanoma cell motility
Source: PLoS One. 2017 Aug 23;12(8):e0183689. doi: 10.1371/journal.pone.0183689 (PMC5568409; doi:10.1371/journal.pone.0183689)
Supplement: S1 Table — (DOCX) [file pone.0183689.s001.docx]

S 1

| Genes | Primers | Sequences |
| --- | --- | --- |
| CD147 | F | 5’ -TAGGATCCATGGCGGCTGCGCTGTTCGTG |
|  | R | 5’- CAGAATTCGGAAGAGTTCCTCTGGCGGACGTTCTTGCC |
| CD147_D34–87 | F | 5’–CACTACCGTAGAAGACCTTGTCTTCCTCCCCGAGCCC |
|  | R | 5’ -GGCTCGGGGAGGAAGACAAGGTCTTCTACGGTAGTG |
| CD147_D105–199 | F | 5’-GCTGCGCACGCGGAGAGGCCCGTGGAGCTGGA |
|  | R | 5’ -CGTGCGCAGCCACCTGAAGCGCCGGAAGCCCG |
| CD147_D207–269 | F | 5’ –CGGGCTTCCGGCGCTTCAGGTGGCTGCGCACG |
|  | R | 5’ -CAGAATTCCTCGTAGATGAAGATGATGGTGACCAGC |
| CD147_D231–269 | F | 5'- TAGAATTCTGACGACGCCGGCGAATGCC |
|  | R | 5’ -CAGAATTCCAGGTGGCTGCGCACGCGGAGCG |
| CD147_D207–269 | F | 5'- TAGAATTCTGACGACGCCGGCGAATGCC |
|  | R | 5'- CAGCTACTCGAGTCACTTTGGATCCTTGGTGGG |

PCR primers
